# Research Progress on the Application of Soda Residue in Cementitious Materials

**DOI:** 10.3390/ma19112228

**Published:** 2026-05-25

**Authors:** Ying Gong, Kaiyue Zhao, Gang Liu, Ying Ba, Yaoyao Wu, Zijian Liu, Yong Yang

**Affiliations:** 1 College of Architectural Engineering, North China University of Science and Technology, Tangshan 063210, China; yinggong0502@163.com (Y.G.);; 2 College of Materials Science and Engineering, North China University of Science and Technology, Tangshan 063210, China

**Keywords:** soda residue, cementitious materials, microscopic properties, macroscopic properties, shrinkage

## Abstract

Soda residue (SR) is an industrial waste produced by the ammonia-soda process. The unique structural characteristics and cementitious activity of soda residue enable it to be used as a mineral admixture for cementitious materials, which is an important way for its resource utilization and also the development direction of green building materials. This paper reviews its potential as a supplementary cementitious material in cementitious materials from physicochemical properties, microstructural influence, and macro performance impact perspectives. Key findings indicate that soda residue enhances cementitious materials’ compactness and early strength through physical filling and chemical activation, yet it concurrently impairs workability and poses durability risks due to chloride content and salt crystallization. An optimized application requires dosage control, chemical modification, and combined use with mineral admixtures. Future research should focus on developing composite binder systems, innovating solid waste-based material preparation, and advancing desalination technologies to enable large-scale, environmentally sound utilization.

## 1. Introduction

**Figure 1:**
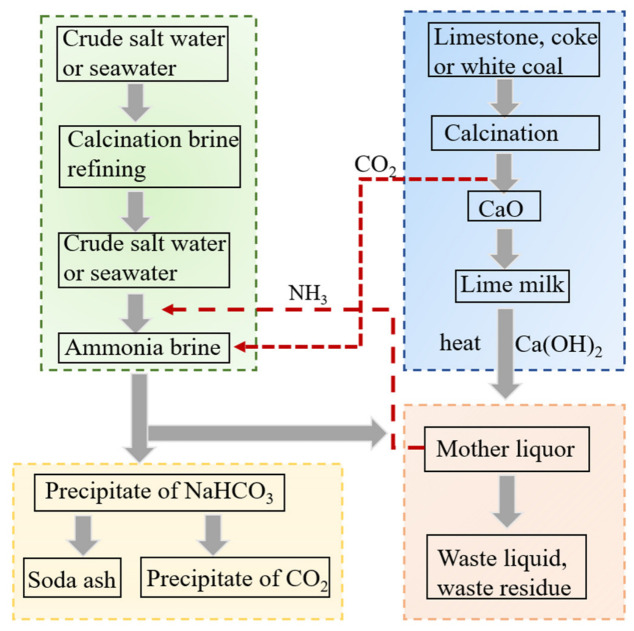
A schematic diagram of soda ash production via the ammonia-soda method [10].

As a commonly used building material, the production and use of concrete have a significant impact on the environment [1]. Traditional production relies on cement, which generates a large amount of carbon dioxide, conflicting with the goals of low-carbon and sustainable development [2,3,4,5]. In the context of pursuing carbon peak and carbon neutrality goals, reducing carbon emissions in the building materials sector has become a critical part of the broader transition toward a green economy, requiring fundamental changes at the material level [6,7]. Therefore, researching new types of concrete is a development trend. Industrial solid wastes such as fly ash (FA), slag, steel slag (SS), and soda residue can serve as supplementary cementitious materials to replace a portion of cement, enhancing concrete performance while promoting resource recycling, aligning with sustainable development requirements [8,9]. By utilizing these industrial by-products, the carbon footprint per unit of concrete can be significantly reduced, contributing to emission reductions across the entire life cycle of buildings. Soda residue is a strongly alkaline by-product of the ammonia-soda process (Figure 1) [10], and its production volume is closely related to the output of soda ash. China emits 7.8–10 million tons of soda residue annually, but its utilization rate in China is only 3–4% [11]. Untreated soda residue not only occupies land resources, leading to land degradation, but also causes soil salinization, posing a threat to plant growth [12]. The large-scale open storage of soda residue has already triggered environmental issues [13]. Furthermore, soda residue contains a large amount of soluble salts, particularly chlorides [14,15] and magnesium salts [16], which can pollute groundwater and threaten human health and the ecosystem [17]. Therefore, the recycling and utilization of soda residue brooks no delay. Incorporating such industrial by-products into low-carbon cementitious material systems offers a promising strategy to address environmental challenges while expanding the technological pathways available for carbon emission reduction.

The porous structure and micro-expansion characteristics of soda residue give it significant advantages in concrete applications. The porous structure of soda residue endows it with superior water absorption and release properties, enabling it to establish a balanced humidity state within the concrete, effectively mitigating cracks caused by autogenous shrinkage and drying shrinkage of the concrete [18]. The micro-expansion characteristics of soda residue can effectively compensate for the shrinkage stress of concrete, inhibit crack development, and thereby enhance the durability of the structure [19]. In addition, its alkali-activated effect can synergize with additives to form a high-performance cementitious system, exhibiting significant advantages in replacing traditional cement-based materials [20]. This paper systematically summarizes the physicochemical characteristics of soda residue and classifies SR according to its pretreatment state. On this basis, it systematically reviews the influence mechanisms of SR on the microstructural characteristics (hydration, pore structure, and microscopic morphology) and macroscopic performance (workability, mechanical properties, durability, and shrinkage) of cement-based materials.

The novelty and original contributions of this review are threefold: (1) Classification by pretreatment state. The effects of SR are systematically classified according to its pretreatment condition (untreated (RSR), water-washed (WSR), wet-ground (WMSR), and calcined (CSR)), providing a reconciling framework for the seemingly contradictory optimal dosages and experimental results reported in the paper. The roles and mechanistic contributions of each SR type across different cementitious matrices are critically differentiated. (2) Critical synthesis of optimal dosages. A structured synthesis table is established that integrates matrix type, SR pretreatment state, replacement level, evaluated properties, and reported optimal ranges, revealing the underlying condition-dependence of reported optimal dosages and explaining why these values cannot be directly compared without explicitly matching their boundary conditions. (3) The Instability of Friedel’s salt (Fs), identifying underappreciated durability risks. This paper critically examines durability concerns that have been previously underexplored, including the long-term stability of Fs under coupled environmental exposures, the fundamental distinction between free and bound chloride, and efflorescence risk, which collectively define the boundary conditions for the safe structural application of SR in cementitious materials. Through this systematic and critical synthesis, this review aims to provide a coherent theoretical foundation for the high-value, safe, and sustainable utilization of SR in the construction materials sector.

In this review, unless otherwise specified, the discussion encompasses SR behavior across paste, mortar, concrete, and alkali-activated cementitious systems.

## 2. Literature Search and Screening Methodology

A systematic literature search was conducted to identify studies on the application of soda residue in cementitious systems. The following databases were searched: Web of Science, Scopus, and CNKI. The search employed the following keyword combinations: (“soda residue” OR “ammonia-soda residue” OR “alkali waste” OR “distiller waste”) AND (“concrete” OR “cement” OR “mortar” OR “paste” OR “cementitious”). The search covered publications from January 2000 to April 2026.

Inclusion criteria were: (1) peer-reviewed journal articles published in English or Chinese; (2) studies directly investigating SR as a component in cementitious materials (including paste, mortar, concrete, and alkali-activated systems); and (3) studies reporting at least one of the following: physicochemical properties, hydration mechanisms, microstructural characteristics, mechanical properties, durability, or shrinkage behavior.

Exclusion criteria were: (1) conference abstracts, dissertations, and non-peer-reviewed reports; (2) studies using SR solely as a soil stabilizer or fill material without discussion of hydration mechanisms; and (3) studies in which SR was not a primary variable (e.g., SR mentioned only as one of many wastes without dedicated analysis).

## 3. Physicochemical Properties of Soda Residue

### 3.1. Physical Properties

**Figure 2:**
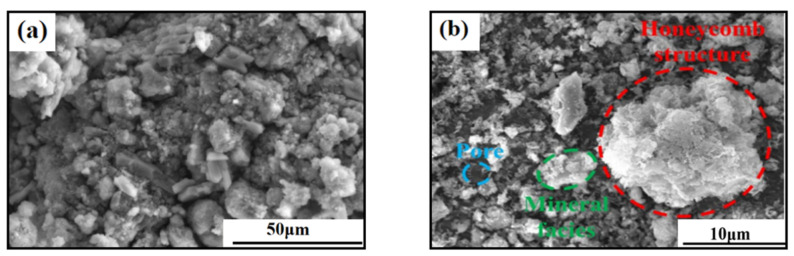
SEM images of soda residue: (**a**) Porous structure [30]; (**b**) honeycomb-like agglomerate [29].

Soda residue exhibits remarkable lightweight characteristics (Table 1) [16,21,22,23,24,25,26,27,28,29] with a porous honeycomb-like microstructure (Figure 2) [29,30], endowing it with high porosity (>60%), high specific surface area (SSA), and small particle size distribution characteristics [16,31,32]. During the concrete mixing process, fine particles of soda residue can be evenly distributed in the cement mix, improving material properties through the following synergistic effects: (1) physical filling, where fine particles effectively fill micro pores, enhancing the compactness of the system [33]; (2) interfacial strengthening, promoting the densification of the aggregate–paste transition zone, thereby improving the mechanical strength of concrete [34]; and (3) internal curing, where porous structure stores and release water, facilitating later hydration [35]. It is worth noting that the high specific surface area of soda residue mainly originated from its unique microstructural characteristics. Cao and Zhang [36] confirmed that soda residue mainly consists of loosely porous aggregates composed of well-crystallized CaSO_4_ and CaCO_3_, with ~50 nm nano-pores significantly enhancing the specific surface area. Wang et al. [37] found that this porous structure comprises nanoscale pores and particle voids within micrometer-sized agglomerates, and the nanoscale pores have a decisive impact on adsorption and reaction activity. Wu et al. [38] further pointed out that the proportion of particles with a diameter of ≤25 μm in the soda residue exceeds 95%. This characteristic distribution of fine particles, coupled with the porous structure, endows the soda residue with excellent surface activity.

**Figure 3:**
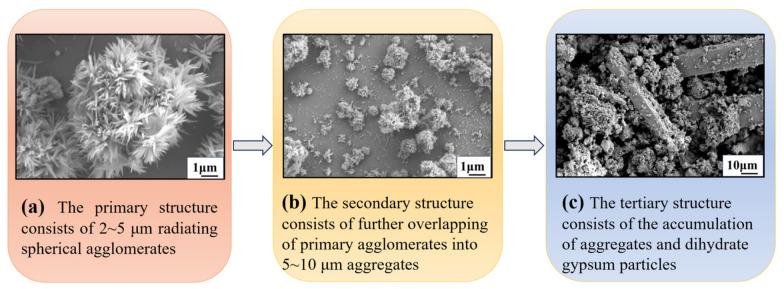
A “three-level model” of the microstructure of soda residue [39].

Meanwhile, Yang et al. [39] established a “three-level model” of the microstructure of soda residue through SEM and TEM analysis: (1) The primary structure consists of 2~5 μm radiating spherical agglomerates formed by the self-stacking of nanoscale calcium carbonate crystals (Figure 3a); (2) The secondary structure is formed by further overlapping of primary agglomerates into 5~10 μm aggregates, with micron-scale through-pores inside (Figure 3b); and (3) The macrostructure is a porous skeleton formed by the accumulation of aggregates and dihydrate gypsum particles (Figure 3c). Cao et al. [40] further confirmed through microscopic characterization that this skeleton is mainly composed of well-crystallized dihydrate gypsum (CaSO_4_.2H_2_O) and CaCO_3_ porous agglomerates, exhibiting significant powder characteristics. It is worth noting that this multi-level assembly structure not only helps to reduce the porosity of concrete but also provides structural support for the soda residue–cement composite system, thereby enhancing the macroscopic properties of the material [41]. In addition, Zhao et al. [42] and Yin et al. [43] observed that the surface of soda residue particles exhibits rough and porous characteristics, and the pore connectivity on the surface and inside of the soda residue particle aggregates is relatively strong. This structural characteristic affects the water absorption performance and mechanical properties of concrete. Meanwhile, Bai et al. [44] found that the honeycomb structure of the soda residue is gradually filled with cementitious products after the hydration reaction, and the structural compactness is enhanced.

### 3.2. Chemical Properties

**Figure 4:**
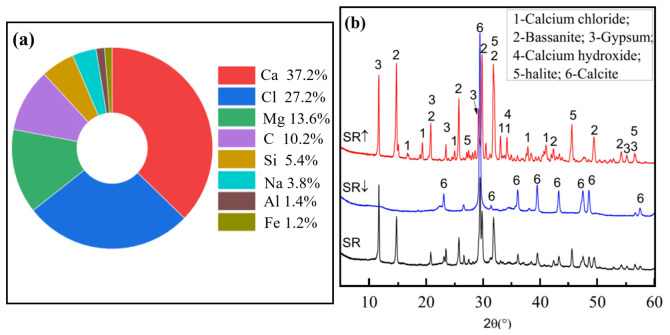
Composition of soda residue (SR): (**a**) Element content of soda residue (all percentages are expressed as mass percent (% *w*/*w*)) [45]; (**b**) XRD patterns of supernatant (SR↑), precipitate (SR↓), and SR [22].

The chemical composition characteristics of soda residue have become a key factor in analyzing its reaction mechanism in cement-based materials and optimizing its application in building materials. Yang et al. [45] characterized the element content and chemical composition of soda residue and the results are shown in Figure 4a, where the content of elements such as Ca, Cl, and Mg is significant, and the main components are calcium salts (CaCO_3_, CaSO_4_, CaCl_2_ and Ca(OH)_2_, etc.), with a small amount of oxides such as SiO_2_, MgO, Al_2_O_3_, Fe_2_O_3_ and Na_2_O (Table 2). However, XRD analysis indicated that soda residue itself does not contain Ca(OH)_2_ or CaCl_2_; these components (primarily CaCO_3_) were detected in the filtrate and precipitate after stirring with water (Figure 4b). Furthermore, the study found that increasing the water-to-solid ratio enhances the solubility of Cl_−_, and the solution remains highly alkaline, with a pH value exceeding 11.4 even at high water-to-solid ratios. Their significantly high alkaline characteristics (pH > 10) make them particularly suitable as alkali-activated materials [22].

According to the GB175–2023 [46] standard, the equivalent alkali content of cement should be controlled below 0.6%. However, as shown in Table 2, the alkali content of most soda residue exceeds this limit, which may induce alkali–aggregate reaction. In addition, elements such as Mg^2+^ and Cl^−^ in soda residue have a significant impact on concrete properties. When the Mg^2+^ content is too high, its corrosivity and crystalline expansion effects can lead to significant deterioration in the strength of cement-based materials [47]. The Cl^−^ content (often exceeding 9%) far exceeds the limit of 0.06% specified in the standard GB/T50476-2008 [48]. Microscopic analysis shows that the Cl^−^ are mainly present in the interstices between particles and in the micron-sized pores of CaCO_3_ crystals [39]. High Cl^−^ content may trigger steel corrosion and volume expansion, ultimately leading to concrete cracking [49,50].

The main alkaline components in soda residue are CaCO_3_ and Ca(OH)_2_, whose hydrolysis reaction can create an alkaline environment with a pH of 10~12 [38]. This alkaline medium can effectively stimulate the pozzolanic activity of materials such as fly ash and promote the formation of hydration products, thereby enhancing the mechanical properties of composite cementitious materials [51]. Although CaCl_2_ and CaSO_4_ increase the complexity of soda residue treatment, studies have shown that CaCl_2_ can significantly promote early-stage hydration of cement, enhancing the early strength of cement-based materials [52]. Meanwhile, CaSO_4_ can react with alkaline components to form ettringite (AFt), whose moisture retention properties contribute to the self-curing of concrete [53,54]. It is worth noting that although a CaCl_2_ solution is suitable for antifreeze engineering due to its low freezing point, corresponding anti-corrosion measures must be taken during its application [55].

**Table 2:** Chemical composition of soda residue %.

Literature	CaO	SiO_2_	Al_2_O_3_	MgO	Fe_2_O_3_	SO_3_	K_2_O	Na_2_O	Cl^−^
An et al. [56]	39.20	5.92	1.92	6.73	0.44	5.33	0.31	4.65	35.40
Guo et al. [21]	43.20	9.87	3.25	9.77	0.91	5.57	0.29	3.93	23.00
Qi et al. [57]	40.30	9.02	2.09	10.00	0.59	6.41	0.31	3.58	27.40
Cheng et al. [58]	52.25	4.06	1.76	2.33	1.17	16.97	0.15	2.46	18.39
Pang et al. [59]	43.51	6.48	1.77	6.91	0.69	7.63	0.47	0.1	32.25
Xu et al. [60]	52.88	10.19	3.25	8.35	1.23	8.87	0.38	1.84	11.95
Li et al. [61]	42.70	9.67	3.45	9.56	0.94	0.11	030	3.84	24.00
Chen et al. [62]	44.70	10.10	2.55	8.09	0.89	5.97	0.43	4.40	20.30
Song et al. [63]	35.12	4.17	2.06	2.97	0.29	2.8	0.33	13.04	39.11

Note: SO_3_ refers to the total sulphate content in cement; all percentage values in Table 2 represent mass percent (% *w*/*w*).

### 3.3. Fabrication Method

To overcome the application bottlenecks of raw soda residue, namely its excessive chloride content, low reactivity, and high compositional variability, researchers have developed various pretreatment strategies. By selectively modulating key properties such as chloride concentration, specific surface area, and mineralogical composition through physical, chemical, or thermal pathways, these treatments fundamentally alter the functional role and behavioral characteristics of soda residue in cementitious systems.

#### 3.3.1. Raw Soda Residue (RSR)

Raw soda residue (RSR) refers to the waste material collected directly from the discharge outlet of the ammonia-soda process without any subsequent industrial treatment. Its characteristics directly inherit the final state of the alkali production process. The primary advantages of RSR are its low processing cost and operational simplicity; however, its inherent drawbacks, namely, high chloride content, substantial compositional variability, and limited reactivity, severely restrict its scope of application.

From a property perspective, the principal concern with RSR lies in its high soluble salt content, particularly Cl^−^. When RSR is incorporated into concrete as a cementitious component, a large quantity of Cl^−^ dissolves into the pore solution, far exceeding the critical chloride threshold required to maintain steel passivation. This poses a fundamental risk of reinforcement corrosion, which represents the most critical barrier to its large-scale utilization [49]. Furthermore, RSR particles exhibit irregular morphology and a wide particle size distribution. Although these particles can provide a limited micro-filler effect, they tend to increase the water demand of the mixture, thereby adversely affecting workability. In addition, the primary mineral phases in RSR (such as Ca(OH)_2_ and CaCO_3_) possess negligible pozzolanic activity. When RSR is used directly as a cement replacement, its contribution to strength development is minimal and may even cause strength loss due to a dilution effect [60].

#### 3.3.2. Washed Soda Residue (WSR)

Water washing is currently the most widely adopted and effective method for removing soluble salts, particularly chlorides from soda residue. This process involves mixing the residue with water, mechanical agitation, and subsequent solid–liquid separation, whereby a substantial fraction of soluble salts such as NaCl and, to a lesser extent, CaCl_2_ are dissolved and removed, thereby yielding washed soda residue (WSR) with significantly reduced chloride content.

The core function of water washing lies in the purification of the chemical composition. Studies have shown that thorough washing can reduce the chloride content of soda residue by more than 90%, rendering it suitable for use as a mineral admixture in ordinary Portland cement concrete. When the chloride content of WSR falls below a critical threshold (0.30%), its incorporation at recommended replacement levels (10%) does not induce discernible reinforcement corrosion. Under these conditions, WSR can partially substitute cement or fly ash to produce concrete that meets both strength and workability requirements [49]. Moreover, the washing process also removes a portion of water-soluble sodium salts, which moderately lowers the pH and reduces the alkalinity of WSR, which is beneficial in mitigating its potential to promote alkali–aggregate reaction (AAR).

#### 3.3.3. Wet-Milled Soda Residue (WMSR)

Wet-milling modification is a physical refinement method in which soda residue is co-ground with water, typically in a ball mill or similar equipment. This process employs mechanical forces to break down agglomerates and reduce particle size, thereby producing wet-milled soda residue (WMSR) with substantially enlarged specific surface area and enhanced reactivity.

Studies have demonstrated that incorporating WMSR as a nucleation seeding additive in Portland cement yields pronounced benefits. The nano or micron-sized WMSR particles provide abundant heterogeneous nucleation sites for the precipitation of cement hydration products, greatly accelerating the precipitation and growth of C–S–H gel. This in turn substantially shortens setting time and significantly boosts early-age compressive strength (a 1.69–fold increase in 12–h strength) [57,64]. Moreover, these fine particles exert a micro-filler effect that refines the pore structure and densifies the hardened cement paste. A notable advantage of wet-milling is that the process is relatively mature and requires no chemical additives during grinding, thereby avoiding secondary pollution. Its principal drawback, however, is high energy consumption. Grinding soda residue to the nanoscale demands considerable electrical energy, which, to a certain extent, limits the economic viability of its large-scale application.

Moderate grinding helps to enhance the activity of soda residue and maintain the integrity of its agglomerate structure [36]. Zhang et al. [65] ground the soda residue and incorporated it into concrete, finding that a 10% soda residue content could significantly reduce the internal micropores of the concrete, enhancing its compactness and wear resistance. Water washing can effectively remove soluble chlorides (such as CaCl_2_, NaCl), but it may result in the loss of active ingredients. Recently, Yang et al. [64] achieved selective leaching of ions from soda residue through a wet grinding process. Their XRD and TG analysis results indicated that this process promoted the dissolution of SO_4_^2−^ and Cl^−^, leading to the weakening of characteristic peaks of CaCO_3_ and Ca(OH)_2_, while the production of NaSO_4_ increased. XPS results further confirmed that wet grinding treatment reduced the binding energies of S, Cl, and Ca, verifying the dissolution of crystal phases such as CaSO_4_·2H_2_O, Ca(OH)_2_ and CaCO_3_ in soda residue.

#### 3.3.4. Calcined Soda Residue (CSR)

The effectiveness of calcination modification depends to a large extent on the calcination temperature. If the temperature is too low, decomposition remains incomplete, and the activation effect is limited. Conversely, excessively high temperatures may induce sintering of the activated phases (CaO), leading to a reduction in specific surface area and a consequent decline in reactivity. Identifying the optimal calcination temperature is therefore of critical importance. As the calcination temperature increases, soda residue undergoes a significant structural evolution process, with its nano-scale porous structure gradually being destroyed and its specific surface area decreasing. Meanwhile, a gradual increase in pore size is evident. At the same time, the agglomerates’ densification degree increases, their particle size distribution concentrates in the range of 0.01 mm to 0.074 mm, and the pore morphology transitions from interconnected pores to large explosive pores [40]. Thermal analysis reveals that moderate calcination (600~800 °C) can effectively retain the active component Ca(OH)_2_, promoting its hydration reaction with pozzolanic materials (e.g., FA, Ground Granulated Blast Furnace Slag(GGBS)) to generate high-strength hydrated calcium silicate [38]. When the temperature exceeds 1200 °C, the active component becomes inactive and is accompanied by an increase in free calcium oxide (f–CaO) content, requiring a combination with a water quenching process to restore its activity [66,67]. It is worth noting that during calcination at 600 °C, the surface of soda residue particles undergoes melting, forming a porous solid structure with a structural framework function, significantly enhancing the mechanical stability of the material [40].

#### 3.3.5. Chemically Modified Soda Residue

Chemical modification refers to a class of methods in which one or more chemical reagents are added to soda residue, or the residue is blended with other industrial wastes, with the aim of altering its chemical composition and reaction characteristics. The objectives of these approaches are diverse and include further reduction in chloride content, activation of latent reactivity, or the generation of synergistic effects with co-existing components.

A more prevalent form of chemical modification is synergistic activation, in which soda residue is combined with other industrial wastes to form a novel cementitious system. Wang et al. [68] blended soda residue with carbide slag (CS) and demonstrated that these two materials supply a substantially higher concentration of OH^−^, generating a “synergistic alkali–calcium activation” effect that powerfully mobilizes the reactivity of slag and enables the production of high-performance, clinker-free cementitious materials. Within such a system, Cl^−^ and SO_4_^2−^ originating from the soda residue can react with Ca^2+^ and (Al(OH)_6_)^3−^ to form Fs and AFt. The mutual filling and interlocking of these hydration products create a dense, high-strength microstructure. The key advantage of this synergistic modification strategy lies in its principle of “treating waste with waste”; it realizes the resource recovery of multiple solid wastes and often generates a synergistic effect where the combined performance exceeds the sum of individual contributions. Nevertheless, chemical modification also introduces new challenges, including the cost of chemical reagents, the increased complexity of mix design for multi-component systems, and the potential introduction of additional impurities.

To more clearly illustrate the regulatory effects of different modification methods, Table 3 provides a comprehensive comparison of the five primary processes.

In summary, the optimization of the microstructure of soda residue is mainly achieved through the following mechanisms: (1) High-temperature treatment promotes the melting and consolidation between particles, forming a denser agglomerated structure [69]; (2) Preparing nano-micron soda residue seed crystals by wet grinding method significantly improves the pore structure, reduces the porosity and critical pore size, and increases the content of gel pores [64]; and (3) Supplementing alkaline activators promotes the participation of active components in the soda residue in the polymerization reaction, and improves the composition and structure of the gel phase [63,70,71].

## 4. Soda Residue Effect on Concrete Micro Characteristics

### 4.1. Cement Hydration

#### 4.1.1. Soda Residue–Cement Binary System

**Figure 5:**
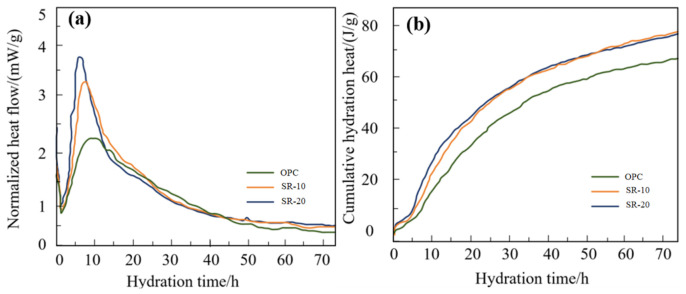
Hydration flow (**a**) and cumulative hydration heat curve (**b**) of the cement–soda residue binary cementitious material system at 72 h (OPC refers to ordinary Portland cement) [23].

The hydration mechanism of the binary system of soda residue–cement can be divided into five stages (Figure 5a) [23]: (1) The rapid exothermic reaction: The cementitious material reacts rapidly with water and releases heat, which is primarily caused by the dissolution of raw material minerals in the liquid phase. The addition of SR significantly increases the exothermic heat of this initial stage, indicating that SR accelerates the dissociation of raw material particles, with higher SR amounts leading to faster dissociation rates. (2) The dormant period: As the reaction progresses, the main hydration products begin to form, and Calcium Silicate Hydrate(C–S–H)/Calcium Aluminate Hydrate(C–A–H) initially fill the pores. (3) The acceleration stage: Raw materials release more heat. The presence of SR causes the fast exothermic peak to become narrow and advances the maximum exothermic point. The gel phase became denser, and its strength jumped. (4) The deceleration stage: The reaction rate gradually slows down, and the hydration products interweave to form a dense microstructure. (5) The steady stage: The reaction tends towards equilibrium, and the rapid precipitation of Fs forms a dense barrier layer on the surface of unreacted particles, creating a barrier effect. It impedes the diffusion of reactants and restricts the further formation of Calcium Aluminosilicate Hydrate(C–A–S–H) gel and restricts the release of Cl^−^, thereby exerting an ion–fixation effect. The structure is optimized, and the porosity decreases. Although excessive dosage (20%) increases the early hydration heat, the high cement substitution rate limits the total amount of hydration products in the later stage (Figure 5b) [23].

In the SR–cement binary system, the regulatory mechanisms governing hydration kinetics differ fundamentally depending on the type of soda residue employed.

Ionic acceleration effect is primarily associated with RSR. RSR, the core mechanism driving hydration acceleration, lies in its ionic effect. The abundant soluble chloride salts (CaCl_2_, NaCl) and Ca(OH)_2_ present in RSR dissolve rapidly upon contact with water, releasing substantial quantities of Cl^−^, Na^+^, Ca^2+^, and OH^-^ into the liquid phase. This process accelerates hydration at three distinct levels: (1) It enhances the ionic strength and electrical conductivity of the pore solution, thereby accelerating the surface dissolution kinetics of the cement clinker minerals (Tricalcium Silicate (C_3_S)) and Dicalcium Silicate (C_2_S)) [60]; (2) CaCl_2_, a well-established cement accelerator, reacts with Tricalcium Aluminate (C_3_A) to form Fs (3CaO·Al_2_O_3_·CaCl_2_·10H_2_O), consuming the initial hydration layer on the C_3_A surface and exposing fresh reaction interfaces [72,73,74]; and (3) elevated Ca^2+^ supersaturation promoting C–S–H nucleation. The high concentration of dissolved Ca^2+^ raises the degree of supersaturation with respect to C–S–H, effectively lowering the nucleation barrier and facilitating heterogeneous precipitation of C–S–H gel on available particle surfaces [75].

Physical nucleation effect, WMSR: For WMSR produced through wet grinding treatment, the dominant mechanism promoting hydration shifts from an ionic effect to a physical nucleation effect. The wet-milling process refines the SR particles to the nano-/micron-scale (D_50_ < 1 μm) and drastically increases their specific surface area [64]. When uniformly dispersed in the paste, these ultrafine particles serve as heterogeneous nucleation substrates for the precipitation of C–S–H gel, substantially lowering the nucleation barrier for hydration products. The study by Yang et al. [64] provides direct evidence for this mechanism: the incorporation of only 6% WMSR increased the 12-h compressive strength of Portland cement by a factor of 1.69, reduced the total porosity from 34.95% to 29.19%, and sharply decreased the critical pore diameter from 155.67 nm to 21.29 nm. That such pronounced effects can be achieved at a remarkably low dosage stands in clear contrast to RSR, which requires a dosage in excess of 10% to produce comparably significant effects, a distinction that clearly reveals the fundamentally different mechanisms of action between the two types.

Reactive calcium effect, CSR: When CSR is produced by calcination at 600~800 °C, its principal mineral, CaCO_3_, undergoes partial decomposition into reactive CaO, while Ca(OH)_2_ also dehydrates to form CaO [38]. This reactive CaO hydrates rapidly upon contact with water to generate Ca(OH)_2_, thereby contributing not only directly to the formation of hydration products but also enhancing the alkalinity of the system and accelerating the hydrolysis of C_3_S and C_2_S. This represents a combined mechanism of chemical and physical activation. It should be noted, however, that excessively high calcination temperatures (beyond approximately 1100~1200 °C) cause CaO crystal sintering and densification, commonly referred to as “dead-burning” (over-burning), which drastically reduces its specific surface area and hydration reactivity [66,67].

#### 4.1.2. Soda Residue–Solid Waste Multi-Component System

SR, as the core component of the multi-component solid waste collaborative technology, utilizes its chemical properties rich in alkali metal oxides and silicate minerals (providing active components and strong alkali environment), porous rough surface expansion reaction interface, and suitable particle size guarantee uniformity to synergistically activate the cementitious system with solid wastes such as ground-granulated blast–furnace slag [56,73], fly ash [20,76], and carbide residue [72,77]. Table 4 shows that soda residue can stimulate the potential activity of mineral admixtures in binary systems, promote the generation of dense microstructures from multi-component hydration products, provide a structural basis for high-strength alkali-activated cementitious materials, and significantly improve the mechanical strength of concrete [61,63]. Table 5 further reveals that the multi-component system constructed by soda residue synergistic slag, iron tailings, and carbide residue forms a multi-dimensional reaction network through ion exchange and network polymerization, and other multi-component physical and chemical coupling, constructing a multi-level pore structure, ultimately achieving a significant improvement in the performance of cementitious materials and the efficiency of solid waste resource utilization.

Although Table 4 and Table 5 systematically document the synergistic mechanisms of SR with single and multiple mineral admixtures, a critical horizontal comparison reveals distinct design philosophies and inherent trade-offs among the three multi-component systems: (1) SR–slag–gypsum: achieves the highest densification through synergistic C–S–H–AFt–Fs network formation, but its high sulfate content raises concerns regarding delayed AFt formation under wet–dry cycling; (2) SR–GGBS–iron tailings: benefits from the additional physical filling of tailings particles with sustained strength development, yet its long-term chloride binding stability remains unverified beyond short-term laboratory tests; (3) SR–carbide slag–red mud–fly ash: uniquely combines Fs formation with red mud adsorption for dual chloride immobilization and excellent early strength, but its quaternary complexity poses significant challenges for mix design standardization and field quality control.

These three systems respectively embody “densification-first”, “filler-reinforced” and “chemical immobilization-first” design strategies. Their divergent strengths and limitations underscore that system selection must balance multiple criteria (early strength, chloride binding capacity, long-term volumetric stability, and practical workability), rather than relying on compressive strength alone.

**Figure 6:**
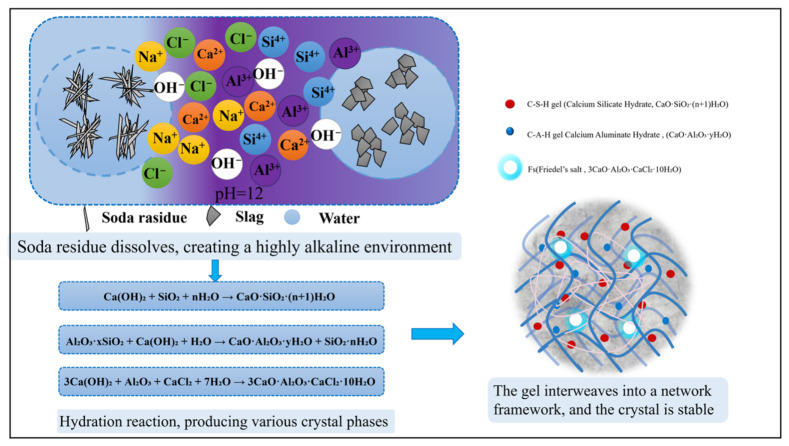
Microstructural evolution of soda residue–slag system.

In summary, soda residue has dual functions of physical filling and chemical activation in alkali-activated systems. The chloride and hydroxide contained in it deeply affect the microstructure evolution path by regulating the concentration of liquid-phase ions and participating in hydration reaction (Figure 6): soluble alkaline substances (NaCl, CaCl_2_, Ca(OH)_2_) dissociate and release Na^+^ and Ca^2+^ upon contact with water to form a highly alkaline environment, directly activating the activity of slag and other additives [84]; at the same time, it promotes pozzolanic reaction of fly ash and slag to generate characteristic crystal phases such as C–S–H, C–A–H, C–A–S–H amorphous gels and Fs, significantly enhancing chemical cementation [57,85]; finally, the gel and crystals interweave to form a spatial network skeleton, and the amorphous phase transforms into a stable crystalline phase, driving the microstructure to mature and stabilize [84].

Numerous studies have demonstrated that raw soda residue (RSR) can be directly employed as an activator for the production of alkali-activated slag cement (AASC) systems: (1) Provision of an alkaline environment: Upon dissolution, RSR releases OH^−^ at concentrations sufficient to disrupt the glassy network structure of slag, inducing its depolymerization [26]. (2) Provision of a calcium source: The Ca(OH)_2_ and CaCO_3_ constituents present in RSR supply Ca^2+^ to the system. As Ca^2+^ is an essential cation for the formation of C–S–H and C–A–S–H gels, this dual “alkali–calcium” action yields an activation effect superior to that achieved with sodium-based alkaline activators alone [63]. (3) Participation in the formation of hydration products: The Cl^−^ and SO_4_^2−^ ions originating from RSR can combine with aluminate phases in the hydration products to form Fs and AFt [63]. These hydration products not only contribute to strength development but also effectively immobilize Cl^−^, reducing its free concentration in the pore solution and thereby mitigating, to a certain extent, the deleterious effects of chloride ions.

In AASC systems, the synergistic performance between soda residue and other components is a complex process. Although the application of RSR embodies the principle of “treating waste with waste”, it also introduces new challenges. The first concern is workability: (1) The strong alkalinity and fine particle characteristics of RSR typically lead to high viscosity and poor fluidity of AASC pastes, posing difficulties for construction operations [25]; (2) volumetric stability: the formation of AFt and Fs is accompanied by volume expansion, while a moderate degree of expansion can fill pores, excessive expansion can induce structural cracking [86]; (3) long-term durability issues, including the risk of alkali–silica reaction (ASR) and carbonation stability, all of which require in-depth investigation [87].

The application of all-solid-waste cementitious systems not only eliminates the carbon emissions associated with cement production but also enables the resource recovery of multiple industrial waste streams, representing an important direction for the future development of building materials. However, the mix design of such systems is exceedingly complex, imposing stringent requirements on the chemical composition and reactivity of the raw materials. Furthermore, their long-term performance, particularly with respect to carbonation resistance and sulfate attack resistance, awaits validation through further engineering practice.

### 4.2. Pore Structure

Soda residue optimizes the pore structure of concrete through physical filling and chemical activity, significantly reducing total porosity, refining pore size distribution, and improving pore morphology and connectivity, thereby enhancing material mechanical properties and durability [88,89,90,91]. This effect is regulated by the dosage of soda residue, the type of cementitious system, and the synergistic effect of multiple materials [88,89,90,91].

**Figure 7:**
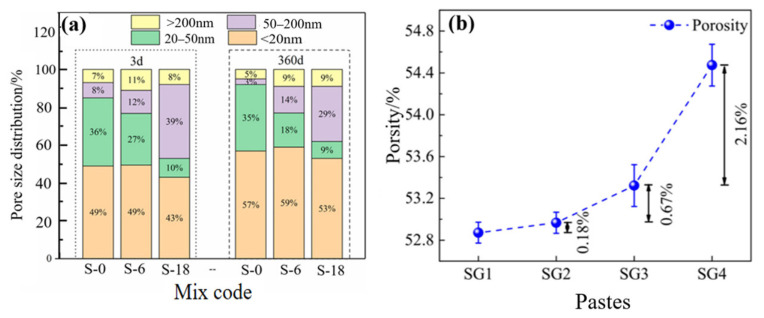
Porosity and pore size distribution of soda residue multi–component system: (**a**) cement mortar with soda residue (no pretreatment) [23]; (**b**) SR–GGBS–cement system at 28 days [22].

Within the low dosage range, the pore structure refinement afforded by soda residue is pronounced; however, the underlying optimization mechanism varies with the SR type. Initially, Xu et al. [23,60] demonstrated through MIP analysis that when the RSR dosage is maintained below 6%, the cumulative pore volume of the hardened paste reaches its minimum, and the proportion of innocuous pores (<20 nm) increases significantly (Figure 7a). This is caused by the synergistic effect of physical filling (micro particles filling the gap to reduce the initial porosity [92]) and chemical coagulation (stimulating the generation of hydration products such as C–S–H gel, AFt and Fs, filling pores and reducing connectivity [93]). Building on these findings regarding porosity reduction, the research of Yang et al. [64] shows that adding 6% wet grinding soda residue reduces the total porosity from 34.95% to 29.19%, and the critical pore size sharply reduces from 155.67 nm to 21.29 nm, the pore size pattern of the cement mortar with SR seed was significantly shifted to the left, with most of the pore size within 50 nm, This effect originates from the nucleation seeding action of WMSR, which promotes the formation of a more uniform and denser C–S–H gel, together with the filling of submicron pores by the nano-sized particles. Furthermore, multi-system validation shows that soda residue can refine pore size distribution in increasingly complex matrices. Lin et al. [22] obtained the optimal pore structure (Figure 7b) in the alkali-activated slag system (soda residue: GGBS = 16:84), the population of large pores was minimized, the pore structure was optimized, and the compressive strength reached its highest value. In this system, the pore refinement arises from two contributions: the GGBS hydration activated by RSR generates C–S–H and AFt that fill the pores, while the appropriate proportion of GGBS supplies sufficient aluminate phases to bind Cl^−^ as Fs, thereby avoiding the negative effects of excess chloride. Xu et al. [94] optimized the pore size distribution and early strength improvement in the soda residue–steel–slag system, revealing the intrinsic relationship between pore structure and durability. Soda residue reduced harmful pore volume fraction, resulting in a decrease in Cl^−^ diffusion coefficient and improved impermeability.

**Figure 8:**
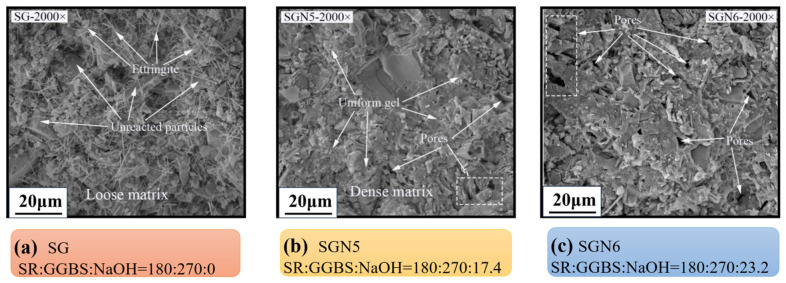
SEM images of 28 days multi-component system [26].

However, there is a critical threshold for the optimization of pore structure by soda residue, and excessive addition will lead to negative effects. Lin et al. [22] found that when the RSR dosage was increased from 8% to 32%, both the total porosity and water absorption rose continuously, and the MIP study by Xu et al. [23] confirmed that the proportion of harmful pores significantly increases when the RSR content exceeds 10%. The mechanism is attributed to the increase in liquid phase ion strength caused by high soluble salts, which leads to flocculation, excessive inert components (CaCO_3_/SiO_2_) diluting the cementitious material to hinder densification [95,96,97], and Cl^−^/SO_4_^2−^ inducing excessive AFt generation (crystal pressure deteriorates pore structure) [98]. Although the densification effect of WMSR at low dosages is markedly superior to that of RSR, its dosage threshold is correspondingly lower. This is because the ultrafine WMSR particles require more water for wetting; at high dosages, non-uniform dispersion may lead to local agglomeration, which paradoxically undermines the filler effect [64]. Meanwhile in the RSR–GGBS system, excessive RSR dilutes the GGBS and reduces the formation of C–S–H gel. Simultaneously, the excess Cl^−^ cannot be fully bound by the available aluminate phases, leading to an elevated free chloride concentration. The resulting imbalance between the Fs and C–S–H gel fractions reduces the stiffness of the matrix [94]. The SEM results of Zhang et al. [26] showed a significant increase and uneven distribution of AFt crystals in the high alkalinity system, with an increase in unreacted particles and large pores (Figure 8). In addition, the dissolution recrystallization process of Fs in high salt environments can generate volume expansion stress, damage pore structure, and reduce strength [94], so it is necessary to avoid such environmental applications.

### 4.3. Microscopic Morphology

**Figure 9:**
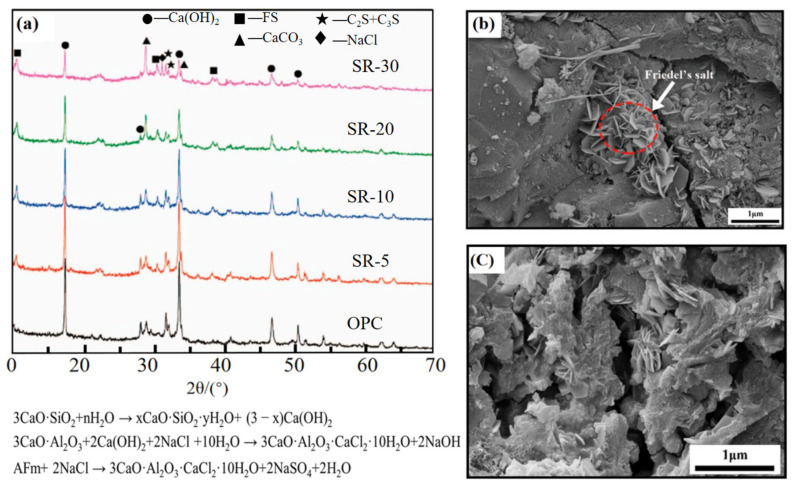
Soda residue–cement binary system: (**a**) XRD patterns of hydration products of produced soda residue–cement at 28 days (OPC refers to ordinary Portland cement) [23,63]; (**b**) SEM image of Fs [23]; (**c**) SEM–EDS analysis of hydration products from cement pastes at curing ages of 28 days [63].

The active components such as Ca^2+^, SiO_2_, and Al_2_O_3_ in soda residue are reconstructed into the microstructure of cement-based materials through secondary reactions: (1) Product phase transition, promoting the formation of new phases such as calcium chloroaluminate (3CaO·Al_2_O_3_·CaCl_2_·10H_2_O namely Fs) and calcium chloride silicate sulfate (Ca_10_(SiO_4_)_3_(SO_4_)_3_Cl_2_) [37], while reducing the content of Ca(OH)_2_ and AFt (Figure 9a) [23,63]. (2) Morphological regulation: SEM showed that Friedel salt (flower like) (Figure 9b) Ca_10_(SiO_4_)_3_Cl_2_ (layered) and other characteristic phases interlaced with C–S–H gel to form a dense complex (Figure 9c) [37,63]; at the kinetic level, CaCl_2_ in soda residue accelerates the dissolution and hydration rate of C_3_S and C_2_S [52,64,99], and its nanoscale size and high specific surface area provide rich reaction interfaces [100]. In terms of microstructural evolution, at the microscopic level, Friedel salts and Ca(OH)_2_ synergistically fill C–S–H pores, constructing low porosity dense structures, ultimately improving mechanical strength and durability [23,101].

Soda residue significantly changes the morphology of hydration products by regulating the chemical environment and reaction process of the cementitious system. In Portland cement, C–S–H is amorphous flocculent or fibrous, while Ca(OH)_2_ is hexagonal plate-like [102,103]. After adding soda residue, the C–S–H structure of the Portland cement system transforms into a honeycomb-like pore-filling structure [23], while in the high alkali-activated slag system, C–S–H evolves into a dense, thin sheet or foil-like stacked morphology [86]. Beyond these morphological changes in individual phases, the synergistic interaction among multiple hydration products has been increasingly recognized. Xu et al. [60] observed that needle-like AFt and flake Fs were interpenetrated in the C–(A)–S–H gel matrix in the soda residue–slag–steel slag–desulfurization gypsum quaternary system, wherein AFt crystal played a role of micro reinforcement, and Fs fixed some harmful Cl^−^. The XRD pattern confirmed that the characteristic peaks of C–S–H, AFt and Fs matched the standard spectrum, revealing the synergistic enhancement mechanism of heterogeneous products. Jiang et al. [104] observed that needle-like AFt and flake Fs were interpenetrated in the C–(A)–S–H gel matrix in the SR–GGBS–phosphogypsum(PG) ternary system, wherein AFt crystals acted as micro-reinforcement, and Fs immobilized harmful Cl^−^. The XRD pattern confirmed that the characteristic peaks of C–(A)–S–H, AFt and Fs matched the standard spectra, revealing the synergistic enhancement mechanism of heterogeneous products. Additionally, the dense microstructure with reduced porosity further accounted for the superior mechanical performance, where the highest proportion of mesopores (<50 nm, 67.62%) and the lowest total pore volume (0.121 cm^3^/g) were achieved at the optimal mix proportion (S10G81P9), correlating well with the 28-day compressive strength of 46.15 MPa.

Soda residue can significantly improve the performance of the concrete interface transition zone (ITZ). Soda residue micro particles aggregate on the aggregate surface to provide nucleation sites, promote heterogeneous nucleation of C–S–H gel, reduce the size and orientation of Ca(OH)_2_, and improve the compactness of ITZ [93]. In addition, the soda residue activator improves the alkalinity of the system, accelerates the hydration reaction in the ITZ region, and forms a better structure [82,105]. Xu et al. [23] found that when the soda residue was greater than 10%, excessive Cl^−^ generated Fs and consumed Ca(OH)_2_, resulting in the reduction in C–S–H gel and matrix stiffness. Ren et al. [106] claimed that when the soda residue was greater than 80%, the C–S–H gel was fragmented, the porosity increased, the micro crack propagation path was shortened, the fracture toughness was reduced, and the interfacial bonding strength was significantly deteriorated.

## 5. Soda Residue Effect on Concrete Macro Performance

### 5.1. Working Performance

Because of its unique physical and chemical properties, soda residue has a complex impact on the working performance of concrete. Yang et al. [49] confirmed that the slump of soda residue concrete was significantly reduced under a low water–binder ratio. Zhao et al. [43] found that the fluidity and density of soda residue–fly ash geopolymer mortar decreased with the increase in dosage, while the porosity increased synchronously, which was attributed to the physical water absorption characteristics of soda residue. Liu et al. [107] pointed out that its strong water absorption can enhance water retention, effectively inhibit bleeding during transportation and construction, and improve uniformity. Li et al. [61] and Ren et al. [106] further revealed that when the particle fineness of soda residue is optimized, and the grading is good, it can fill the micro gap and synergistically improve the cohesion and water retention capacity. The effect of soda residue on the workability of concrete is regulated by multiple factors: its alkaline component and CaCl_2_ accelerate the hydration of cement, resulting in early setting, and reduce the fluidity and plasticity [52]; fine particle size increases water demand [44], irregular morphology increases particle friction resistance and inhibits fluidity [22]; and strong water absorption adsorbs free water to reduce lubrication and significantly reduce fluidity [26].

### 5.2. Mechanical Properties

**Figure 10:**
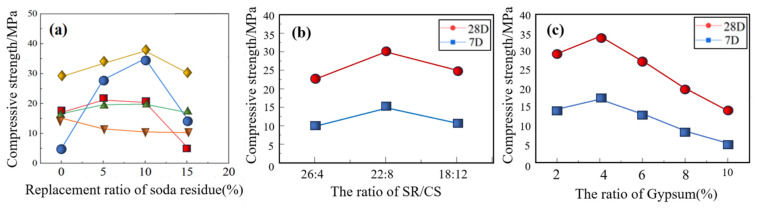
The effect of soda residue on the mechanical properties of multi-cementing systems: (**a**) Compressive strength by soda residue ratio [30,37,108,109,110]; (**b**) compressive strength by soda residue/carbide slag ratio [72]; (**c**) compressive strength by gypsum ratio [72].

Proper addition of soda residue can improve the mechanical properties of cementitious materials. However, when the amount of soda residue exceeds a certain proportion range, the strength of the test piece will decrease (Figure 10a) [30,37,108,109,110,111,112,113]. In cement-based systems, Yang et al. [49] pointed out that the strength can be improved by replacing 10% cement with soda residue, but a water-reducing agent should be added in the case of a low water-to-binder ratio system. Furthermore, in multi-component solid-waste systems (e.g., soda residue–fly ash–slag), similar optimal dosage patterns have been observed. Li et al. [114] showed that the compressive and flexural strength reached the peak when the ratio of soda residue to fly ash was 40:60. Xu et al. [111] found that the strength of the soda residue–fly ash mineral powder mixed system increased first and then decreased when the soda residue content was 20–30%. Building on these findings, Zhang et al. [72] further refined the optimal dosage range in a SR–CS activated GGBS–FA system. They demonstrated that the 28-day compressive strength reached a maximum of 30.4 MPa when the soda residue content was 22%, and the carbide slag content was 8%; increasing the soda residue to 26% or decreasing it to 18% both led to strength reduction (Figure 10b). Moreover, the addition of 4% desulfurization gypsum further enhanced the 28-day strength to 34.2 MPa, confirming a synergistic sulfate activation effect (Figure 10c). Beyond the influence of the soda residue proportion, its physical fineness and activator concentration also significantly affect mechanical performance. Liu et al. [115] further revealed that, among the three factors examined (soda residue-to-fly ash ratio, liquid–solid ratio, and sodium silicate concentration), the sodium silicate concentration exerted the most significant influence on compressive strength, followed by the liquid–solid ratio, while the soda residue-to-fly ash ratio had the least effect. The optimal compressive strength (17.778 MPa) was obtained at a sodium silicate concentration of 3.0 mol/L, a liquid–solid ratio of 1.1, and a soda residue-to-fly ash ratio of 2:3. They also found that reducing the sieving size of soda residue from <2 mm to <0.25 mm enhanced the 28-day compressive strength by approximately five times, and a reduction to <0.5 mm led to an increase of about 51%. In addition, Guo et al. [78,116] showed that the splitting tensile strength and flexural strength of the solid waste-based concrete made of SR–CS–FA cementitious material combined with iron tailings aggregate are equivalent to those of ordinary concrete, which is due to the formation of dense ITZ structure between the cementitious system and iron tailings to reduce interface defects, but the elastic modulus of the system is low and the brittleness characteristics after peak value are significantly increased. Soda residue has dual effects on concrete. When properly mixed, its hydrate forms a compact structure through cemented aggregates and closed pores, which significantly improves the short-term strength. However, the high salt content of excessive soda residue is easy to induce long-term efflorescence and degrade the durability. The optimization strategy includes the use of soda residue–carbide slag wet basis, combined with precise temperature control curing at 60~75 °C for 12 h, which can accelerate the hydration reaction, compact the microstructure, and improve the mechanical properties, but it is necessary to avoid overheating or overtime curing [21,108].

Table 6 summarizes matrix type, soda residue status, substitution level, evaluation characteristics, and the optimal ranges reported, aiming to visually illustrate the variability and patterns of optimal incorporation levels under different study conditions. Synthesizing the data in the table above, we can deeply analyze the key factors influencing the optimal dosage of soda residue from the following two dimensions.

Matrix type is the primary factor determining the optimal dosage range of soda residue, as it fundamentally defines the role that soda residue plays within the system: (1) In Portland cement-based materials, the role of soda residue is “supplementary” or “functional”. As a blending material, its optimal dosage is constrained by the hydration product environment of the cement. The highly alkaline pore solution formed by cement hydration is the core for maintaining the cementitious nature of the system. Excessive soda residue dilutes this environment and introduces large amounts of non-reactive or low reactivity Cl^−^ and SO_4_^2−^, which may disrupt the microstructure of the hardened cement paste and lead to strength reduction. Therefore, its optimal dosage is typically low, such as 6% [64], 10% [49]. (2) In alkali-activated materials (AAMs), the role of soda residue transforms into a “core” or “dominant” one. It can be the primary alkaline activator (e.g., 70% soda residue activating slag [86]) or a cementitious main body alongside activated components such as slag (e.g., 20% soda residue with 80% slag [63]). In such a system, the alkalinity and ionic environment of the entire cementitious system are built around the characteristics of the soda residue, thus substantially raising the upper limit of its dosage. (3) In autoclaved aerated concrete (AAC), the role of soda residue is that of a “raw material”. It provides a CaO source and participates in the autoclave hydrothermal synthesis reaction to generate high-strength crystalline phases, such as tobermorite. The optimal dosage in this case must be precisely matched with the SiO_2_ source (e.g., fly ash) to satisfy the stoichiometric ratio and ensure a reasonable reaction process. A dosage of 20–30% is a commonly reported optimal range [117,118], reflecting its efficiency in providing calcium.

Replacement level itself is not an independent influencing factor; rather, it is a quantitative outcome that directly reflects the “concentration” of soda residue in the system. As discussed above, the optimal replacement level varies dramatically depending on the matrix type: (1) <5%: In high-performance cement-based materials requiring high dimensional stability and long-term durability, the dosage of soda residue is typically restricted to extremely low levels to avoid its adverse effects [37,60]. (2) A range of 5–15%: This is the most common optimal range reported for ordinary Portland cement-based materials (e.g., concrete, mortar, cement–stabilized soil). Within this range, the “micro–aggregate effect” and “pozzolanic effect” of soda residue are fully exerted, effectively filling pores and improving the interfacial transition zone, thereby reducing material costs and enhancing certain early-age properties without significantly compromising long-term performance [63]. (3) A range of 15–40%: This dosage range is more commonly encountered in autoclaved aerated concrete, where soda residue serves as the primary calcareous raw material [117], or in clinker-free cements where it is an important component of the composite cementitious system [119]. Here, the contribution of soda residue extends beyond physical filling; more critically, its chemical components participate directly in the principal hydration or hydrothermal reactions [43]. (4) Greater than 40%: In alkali-activated systems, when soda residue plays the role of an activator, its dosage can far exceed conventional limits. A dosage as high as 70% signifies that soda residue constitutes the main body of the system, with its function shifting from “replacement” to “dominance” [56,86].

**Table 6:** Optimal mixing amounts of variously modified soda residues in different cement-based materials.

Matrix Type	Soda Residue State	Replacement Level	Evaluated Properties	Reported Optimal Range
Autoclaved Sand Aerated Concrete (AAC) [120]	SR (Not specified)	SR replacing lime: 10–30%	Volumetric expansion, dry density, compressive strength	SR replacing lime: 20%
Autoclaved Aerated Concrete (AAC) [118]	Not specified	SR dosage: 0–30%	Compressive strength	Approx. 20–30%
Autoclaved Aerated Concrete (AAC) [117]	Not specified	SR: 20% (of total material)	Dry density, compressive strength	20% (SR + construction waste + lime + cement + gypsum system)
Alkali-Activated Slag Geopolymer [86]	Not specified	SR: 70% (of total mass of precursor)	Compressive strength	70% SR
Clinker-free composite cementitious material [63]	Not specified	SR: Slag = 1:4 (mass ratio)	Compressive strength, microstructure	SR: Slag = 1:4 (SR ≈ 20% of total binder)
Composite Portland cement [23]	Untreated (RSR)	SR: 5%, 10%, 20% (replacing cement)	Pore structure, setting time, compressive strength	<6% (pore structure); 5–10% acceptable; >10% reduces 28 d strength
Alkali-activated slag cement (AASC) [22]	Untreated (RSR)	SR: 8–34% (replacing GGBS)	Compressive strength	SR: GGBS ≈ 16:84 (SR ≈ 16%)
Clinker-free concrete (SR-GGBS-SS-FGD) [60]	Untreated (RSR)	SR: 6% (of total binder)	Compressive strength (up to 360 d)	6% SR (360 d strength 66.31 MPa)
Portland cement concrete [49]	Washed (WSR)	WSR: 10% (replacing cement or fly ash)	Compressive strength, Cl^−^ content	10% WSR when Cl^−^ < 0.3%
Portland cement [64]	Wet-milled (WMSR)	WMSR: 6% (as nucleation seeding additive)	12 h compressive strength, hydration heat	6% WMSR (12 h strength increased by 1.69 times)
Alkali-activated slag geopolymer [121]	Chemically modified (SR + CS)	SR + CS as composite activator	28 days compressive strength	≥27 MPa at 28 days (specific proportion depends on system)
Geopolymer [68]	Chemically modified (SR + PG)	SR + PG as composite activator	Compressive strength, Microstructure Compressive strength, microstructure	Significant synergistic enhancement (AFt + C–S–H)

### 5.3. Chloride-Related Risks

#### 5.3.1. Steel Corrosion

The most immediate and severe challenge posed by introducing SR into concrete is the enormous discrepancy between its chloride content and the limits stipulated in relevant national codes and standards. Table 7 summarizes the permissible limits for chloride ions (as a percentage of cement weight) in concrete, as specified in various standards for typical environmental categories. Cl^−^ in concrete is the primary culprit responsible for inducing reinforcement corrosion. The soda residue is rich in soluble chloride, which significantly increases the concentration of Cl^−^ in the concrete and forms an initial chlorine reservoir [37]. When the critical value is exceeded, it destroys the passive film of the reinforcement and causes corrosion [122,123,124,125,126]. In particular, it accelerates the migration of Cl^−^ in the humidity fluctuation environment, resulting in weakening of the reinforcement section and cracking of the protective layer [23,49], degradation of the bond between steel and concrete, and ultimately compromising the safety and durability of the structure.

To control this risk, stringent limits on chloride ion content have been established in design codes worldwide. The codes require the chloride ion content to be controlled below 0.3%. In contrast, the chloride content of SR generally exceeds 10%. Even when replacing cement at a modest substitution rate of 10%, the chloride ion content in the concrete would instantaneously exceed the 0.3% limit several times over. Lin et al. [22] demonstrated that when SR constituted 8% of the total cementitious material, the free chloride ion content by mass in the hardened cement paste at 28 days remained as high as 0.13%, far exceeding the stringent limit of 0.06%. Although the wet base SR:CS:GGBS:FA (1:0.5:3:0.5) quaternary cementitious material developed by Zhao et al. [43] meets the P.O42.5 strength standard, it is still not suitable for reinforced concrete due to its high chlorine characteristics. In practical application, the soda residue must be strictly controlled in dosage, dechlorination pretreatment or avoiding the reinforcement structure to ensure that the total chlorine content is lower than the specification limit to ensure durability [22,37].

#### 5.3.2. Free Versus Bound Chloride Thresholds

The Critical Chloride Threshold Level (CCTL) is defined as the minimum concentration of free chloride ions in the pore solution required to induce depassivation and initiate corrosion of reinforcing steel under specific conditions. The determination of CCTL is central to corrosion risk assessment and service life prediction. However, extensive research demonstrates that CCTL is not a fixed constant but a complex variable influenced by numerous factors, including material properties [130], environmental conditions, and testing methodologies.

Cement type and mineral admixtures are one of the most significant factors influencing CCTL. For ordinary Portland cement concrete, CCTL is generally considered to range from 0.2% to 1.0% (by weight of cement). However, the situation becomes considerably more complex when substantial amounts of mineral admixtures (e.g., FA, slag) are incorporated. On the one hand, these admixtures can decrease the pH of the pore solution (particularly with high-volume fly ash), weakening the passive film stability and thus lowering the CCTL [130]. On the other hand, they enhance chloride binding capacity through pozzolanic reactions or the formation of hydration products. Zhang et al. [131] noted that reported CCTL values exhibit an extremely wide range of variation, from below 0.1% to over 1.0%, which is closely related to the type and dosage of mineral admixtures involved. Currently, there are few papers specifically addressing the effects on CCTL. However, based on extensive fundamental research and corrosion mechanism studies on CCTL, it can be reasonably inferred that increased temperature reduces the CCTL value, accelerates the corrosion reaction, while the type of reinforcing steel also influences the critical chloride ion concentration [132].

The determination of CCTL itself also faces considerable challenges. Traditional long-term immersion methods require months or even years, failing to meet the demands of rapid assessment [130]. Consequently, researchers have developed various accelerated testing methods. However, different testing methods, criteria (e.g., abrupt change in corrosion potential, current density exceeding a threshold), solution compositions, and experimental setups can all lead to significant discrepancies in the measured CCTL values. Table 8 summarizes the significant variations in CCTL across different studies, providing an intuitive visualization of its uncertainty.

For high-chloride SR concrete, the assessment of CCTL becomes exceptionally difficult. On the one hand, its extremely high initial chloride content means that the reinforcing steel is in a “high-risk” environment from the outset. On the other hand, the complex chemical composition of SR itself (e.g., high pH, potential presence of sulfates) and the instability of Fs make its true CCTL difficult to predict. Simply applying CCTL values derived for ordinary concrete may severely underestimate its corrosion risk.

#### 5.3.3. Limitations of Chloride Binding and Long-Term Stability Concerns

Although the formation of Fs is widely regarded as a form of chloride “immobilization”, this immobilization is not permanent [134]. Under specific physicochemical conditions, the crystal structure of Fs can be disrupted, leading to the release of the once “captive” chloride ions, forming what is termed an “internal source of chlorides”. This dynamic process of “immobilization–release” is key to the long-term performance degradation of high-chloride SR concrete and constitutes its high corrosion risk.

First, chloride ion release induced by sulfate attack, SO_4_^2−^, possesses an ionic radius and charge similar to those of Cl^−,^ and their chemical properties are also relatively close, making them one of the most “effective” ions capable of destabilizing Fs. Studies have demonstrated that when sulfate ions are present in the environment, they undergo an anion exchange reaction with Fs, displacing the chloride ions (Cl^−^) from their structure [135]. The reaction process can be represented as follows:

3CaO·Al_2_O_3_·CaCl_2_·10H_2_O (Fs) + SO_4_^2−^ → 3CaO·Al_2_O_3_·CaSO_4_·10H_2_O (AFt) + 2Cl^−^

The reaction product is AFt, whose structure can accommodate the larger sulfate ion more readily than that of Fs. The essence of this process is that, thermodynamically, sulfate ions exhibit a stronger affinity for the layered AFm phase than chloride ions. Shen et al. [135] elucidated this mechanism at the atomic level: the incorporation of sulfate ions, through its charge redistribution effect, weakens the electrostatic interaction between chloride ions and the (Ca_2_Al(OH)_6_)^+^ layers, and simultaneously disrupts the hydrogen bond network between chloride ions and water molecule layers, thereby substantially lowering the energy barrier for chloride ions to detach from the crystal structure and facilitating their release into the pore solution.

This constitutes a potential internal risk source. Even if concrete successfully immobilizes the majority of chloride ions within Fs at an early stage, once it encounters external sulfate attack (e.g., from seawater, groundwater, or sulfates in soil), these “bound” chloride ions may be “reactivated” into a free state, causing the chloride ion concentration in the pore solution to resurge, thereby posing a persistent threat to the safety of reinforcing steel. When concrete has a high soda residue content, which may introduce sulfate impurities, the threat to the safety of reinforcing steel is enhanced. 

Secondly, changes in alkalinity (pH) affect the stability of Fs [136]. The stable existence of Fs requires a highly alkaline environment. The strong alkalinity of hardened cement paste (with pH typically ranging from 13 to 14) is critical for maintaining the passive film on reinforcing steel and the stability of various hydration products, including Fs [137,138]. However, during the long-term service life of concrete, its internal alkalinity may decrease for several reasons. The first is carbonation. Atmospheric carbon dioxide (CO_2_) reacts with calcium hydroxide (Ca(OH)_2_) in the pore solution to form calcium carbonate (CaCO_3_) and water, leading to a sharp decline in the pH of the pore solution [139]. When the carbonation front reaches the surface of the reinforcing steel, the pH may plummet from above 13 to below 9, which will directly result in the breakdown of the steel’s passive film [140,141]. Meanwhile, acid rain erosion or the metabolic activity of certain microorganisms also consumes the alkalinity within concrete [142]. Therefore, carbonation and neutralization of the concrete cover represent another important cause leading to the loss of the Fs stability and the subsequent re-release of chloride ions.

Wet–dry cycles and temperature variations also affect the stability of Fs and the speciation of chloride ions. In environments with alternating wetting and drying, concrete undergoes repeated cycles of water absorption and moisture loss. During the drying process, salts in the pore solution become concentrated and may exceed the solubility limit of Fs at that temperature, leading to its precipitation from the supersaturated solution. However, this portion of chloride ions may re-dissolve and become free ions upon subsequent water absorption. Furthermore, the drying process generates capillary pressure within the concrete, potentially inducing microcracks that provide rapid pathways for chloride ion transport [143]. Previously “bound” chloride ions may have a higher probability of re-entering the pore solution, increasing the local chloride concentration.

In summary, the “immobilization” of chloride ions by Fs is relative and conditional. Under the combined or sequential action of various environmental factors (including sulfate attack, carbonation, wet–dry cycles, and elevated temperatures), this immobilization may be disrupted, forming a persistent, internal source of chloride ion supply. This “immobilization–release” double effect renders the durability issues of high-chloride SR concrete more insidious and complex. Traditional static assessment methods are inadequate to capture their dynamic risks, and this must be given serious attention.

#### 5.3.4. Limitations for Structural Applications

The limitations of high-chloride SR concrete in structural applications are mainly reflected in the following aspects: (1) Inability to meet mandatory code requirements for total chloride content: As discussed, the chloride content of SR far exceeds code limits. Regardless of the mix design, it is difficult to reduce the total chloride content (both free and bound) in the final hardened body to within safe limits. This renders it unsuitable for direct use in various types of reinforced concrete structures defined by codes, especially those located in humid, outdoor, or corrosive environments [131]. (2) Potential degradation of mechanical properties: The high content of soluble salts and chlorides may affect the hydration process and microstructure of the cement. Some studies have observed that although a certain amount of SR can accelerate early hydration, excessively high dosages may lead to reduced long-term strength or deteriorated durability indicators [22]. This performance uncertainty introduces additional risk to structural design. (3) Unpredictability of long-term performance: Owing to the “immobilization–release” mechanism, the long-term performance of high-chloride SR concrete is not static. Under environments involving carbonation [139], sulfate attack [135], etc., its interior may become a continuous “chloride generator”, causing the risk of reinforcement corrosion to accumulate progressively over time. This unpredictability of long-term evolution contradicts the century-long design service life requirements typical of structures. (4) Strict limitations on applicable scenarios: For the reasons stated above, the application of high-chloride SR concrete must be strictly limited to non-load-bearing, dry, indoor environments free from groundwater erosion. Research has successfully applied it as a lightweight fill, subgrade cushion, or backfill material in road engineering [144]. In these applications, reinforcement is either completely excluded or is situated in an extremely low–risk environment. However, beyond these specific scenarios, its application is accompanied by extremely high structural safety risks.

### 5.4. Durability

#### 5.4.1. Chloride Penetration Resistance Performance

The chloride penetration resistance of soda residue concrete is governed by a dual regulatory mechanism: the interplay between short-term penetration resistance (derived from pore structure optimization) and the long-term risk of chloride re-release (due to Fs instability). The active components in the soda residue fill the micropores by generating dense cementitious products (C–(A)–S–H, AFt, Fs), which significantly improve the chloride ion penetration resistance of concrete [61]. This improvement effect is particularly prominent in the SR–GGBS system, which combines with Cl^−^ to form Fs to solidify free chloride ions, and the binding rate increases with the curing time [22,30]. The comparative study of multiple systems shows that the chloride ion solidification ability of carbide slag is better than that of GGBS and fly ash, and the impermeability can be enhanced by mixing soda residue and carbide slag [108]. Steel slag and soda residue can improve the ITZ compactness and then improve the overall impermeability [145]. The chloride salt in soda residue has a dual regulation mechanism on the chloride ion permeability resistance of concrete. When a certain amount is added, the calcium component reacts with Cl^−^ to form Fs to fix free chloride ions [58,106], and Fs fills the pores to enhance compactness and improve the permeability resistance [108]. However, excessive incorporation will lead to sharp reduction in C–S–H gel content and sharp increase in Fs, which will lead to increase in porosity and structural looseness, and significantly reduce the impermeability [106,107].

#### 5.4.2. Sulfate Corrosion Resistance Performance

Soda residue has a complex impact on the sulfate resistance of concrete. Its alkaline substances promote the generation of hydration products and fill the pores to improve compactness, and enhance the corrosion resistance [25,79]. At the same time, Cl^−^ competes with SO_4_^2−^ to inhibit sulfate attack [62]. However, soda residue changes the formation ratio of AFt and Fs. Fs consumes Ca(OH)_2_, which weakens the buffering capacity of the sulfuric acid environment [30,37]. Excessive AFt causes structural damage and increased porosity due to volume expansion, which significantly reduces the corrosion resistance [29,62].

The impact of soda residue on the sulfate resistance of concrete needs dialectical evaluation. The carbonate composition in the soda residue–cement system is easy to react with sulfate to damage the structure and reduce the corrosion resistance [23], so it is not suitable for high sulfate resistance engineering scenarios. In view of this, soda residue is usually used in combination with other mineral admixtures to improve sulfate resistance. Xu et al. [146] confirmed that the corrosion resistance of the soda residue–GGBS synergistic system is better than that of ordinary Portland cement at an appropriate dosage. Yang et al. [64] performed a 5% Na_2_SO_4_ erosion test showing that the strength and quality of mortar with 20% soda residue content had zero loss. Zhao et al. [147] also found that C–S–H/N–A–S–H generated by the soda residue fly ash system has the ability to repair acid erosion. However, it should be noted that excessive soda residue will weaken sulfate resistance, and the Na^+^/K^+^ contained in it may induce alkali–aggregate reaction [148,149,150].

#### 5.4.3. Efflorescence

Efflorescence refers to the phenomenon where salts dissolved in water migrate to the surface of building materials along with moisture, and upon moisture evaporation, the salts precipitate as crystals, forming a layer of white or colored powdery deposit. For SR–based materials, due to the intrinsic high content of soluble salts (NaCl, Na_2_CO_3_, Na_2_SO_4_, etc.), efflorescence is an apparent quality issue that cannot be ignored in its application.

Although research directly targeting efflorescence in SR-based materials is limited, referencing studies on related waste residues (red mud [151], MSWI fly ash [152]), efflorescence can be suppressed through the following approaches: (1) Reducing the source of soluble salts: Pretreating SR before material preparation, such as water washing, can effectively remove most of the water-soluble Cl^−^ and Na^+^ [49]. (2) Reducing porosity and connectivity: By optimizing mix proportions, lowering the water-to-binder ratio, incorporating ultra-fine mineral admixtures such as silica fume, or employing measures like high-pressure steam curing, the material’s porosity can be significantly reduced, blocking capillary water transport pathways. (3) Incorporating mineral admixtures: Incorporating pozzolanic materials (FA, GGBS) can react with alkaline substances such as Ca(OH)_2_ in secondary reactions, consuming some soluble alkalis and generating C–S–H gel of lower alkalinity, thereby reducing the concentration of alkali metal ions in the liquid phase. (4) Surface treatment: After the material has hardened, applying a hydrophobic agent or waterproof coating can form a protective film on the surface, preventing moisture ingress from the outside and the precipitation of internal salts. Wu et al. [153] found that incorporating phosphogypsum could effectively transform the efflorescence product from Na_2_CO_3_ to Na_2_SO_4_∙10H_2_O, which has lower solubility, thereby mitigating the degree of efflorescence. This concept is equally applicable as a reference for SR-based materials.

#### 5.4.4. Long-Term Performance

Material durability is a dynamic, continuously changing process. Long-term performance refers to the ability of a material to resist various environmental factors and load effects during its service life, maintaining its functionality and safety. For SR-based materials, due to their relatively short application history in the field of civil engineering, the assessment and prediction of long-term performance are key challenges and focal points of current research. Current understanding is primarily based on short and medium-term accelerated tests and theoretical inferences.

When SR is used as a cementitious component, during the initial curing period, due to the strong alkaline activation effect of SR, material strength increases rapidly [83]. In alkali-activated GGBS–FA systems, early strength mainly originates from the rapid hydration of GGBS [108]. However, the development of long-term strength depends on the completeness of the reaction and the stability of the microstructure. Nanayakkara et al. [154] found that AAS (alkali-activated slag) concrete still exhibits some strength gain under long-term curing (1 year), but the rate of increase gradually slows down. Wardhono et al. [155] observed that due to ongoing carbonation or microcrack propagation, the strength of certain AAS concretes may exhibit a slight decrease at later ages (360 days). Although there is a lack of research experiments specifically investigating the long-term performance of SR composite cementitious materials, reasonable inferences can be drawn based on relevant theories and studies of similar materials: (1) Early strength advantage: The alkaline components in the soda residue may accelerate the early hydration reaction, thereby enhancing the 28-day strength [23]. (2) Long-term performance risk: Prolonged accumulation of soluble salts may induce crystallization pressure, compromising the stability of hydration products.

### 5.5. Shrinkage Properties

#### 5.5.1. Self-Shrinkage

The autogenous shrinkage of concrete with a low water–binder ratio results from capillary tension caused by cement hydration [156]. The key to crack control is to inhibit self-shrinkage, which can be achieved through internal curing technology or a shrinkage-reducing agent [157,158,159,160]. The internal curing material can relieve the self-drying effect by continuously releasing water, maintain the pore water saturation state, and reduce the capillary shrinkage stress, so as to significantly inhibit the self-shrinkage [161,162].

Soda residue can significantly inhibit the autogenous shrinkage of concrete through the triple synergistic mechanism: (1) Internal curing effect: Its micro pore structure continuously releases water to maintain internal humidity and reduce capillary stress [18]. (2) Micro-expansion compensation: CaSO_4_ reacts with C_3_A to produce expansive AFt neutralizing contraction [18,19]. (3) Activity regulation: Alkali residue replacing cement reduces early hydration activity and shrinkage source [19]. Pu [35] confirmed that 10–15% content can improve internal humidity and effectively reduce self-shrinkage. Yue [163] further optimized the process and found that 5.25% dechlorinated soda residue can effectively reduce autogenous shrinkage of concrete under the premise of ensuring the strength of 28 days, and the effect of additional underwater synergy is more significant. However, excessive soda residue will damage the mechanical properties, so the dosage needs to be strictly controlled, and the dosage of admixture for its treatment process needs to be further studied [35,163].

#### 5.5.2. Chemical Shrinkage

Chemical shrinkage is an inherent property of the cement hydration process, which results from the reduction in solid volume of hydration products [164,165,166]. Soda residue regulates the chemical shrinkage of cementitious materials through dual mechanisms: (1) Alkali excitation accelerates hydration, the depolymerization and repolymerization of GGBS and steel slag. In the strong alkali environment provided by soda residue, this process accelerates the formation of C–S–H and C–A–S–H, and promotes the centralized completion of chemical shrinkage in a short time [37,60]; microstructure densification reconstructs later shrinkage dynamics [60]. (2) Ion-induced expansion compensation occurs when SO_4_^2−^/Cl^−^ react with C_3_A to form AFt or Fs. Xu et al. [94] confirmed that Fs can compensate for the expansion gap of AFt reduction. Lin et al. [84] verified that AFt and C–S–H cooperatively filled the pores and brought volume expansion effect in the soda residue–carbide slag system. Liu et al. [82] pointed out that the soda residue activator can realize contraction/expansion directional switching.

Based on the dual role of soda residue as alkali activator and expansion source, under the appropriate dosage and compound conditions, it can effectively control chemical shrinkage and significantly improve the volume stability of concrete by accelerating hydration densification and expansion phase volume compensation. It is necessary to further quantify the expansion–contraction equilibrium point of the composite system to optimize the long-term performance.

#### 5.5.3. Drying Shrinkage

The drying shrinkage of concrete is closely related to the pore structure of cement paste [167]. The higher the total porosity or the larger the proportion of macropores (>50 nm), the more significant the drying shrinkage of cement-based materials [168,169]. Drying shrinkage can be effectively inhibited by refining pore size and reducing pore connectivity [170,171]. Ning et al. [172] found that in the concrete strengthened by multiple solid wastes, with the increase in the amount of waste residue (including soda residue), the hydration product C–S–H gel/AFt continued to fill the pores, driving the microstructure to evolve from a three-dimensional network to a dense body, thus enhancing its ability to resist external interference. However, Guo et al. [78,116] found that although solid waste concrete has high strength, its elastic modulus is lower than that of ordinary concrete of the same grade. This low modulus characteristic gives the material excellent “stress absorption” ability, and can produce greater deformation without causing excessive stress under constraint conditions, significantly reducing the risk of cracking caused by drying shrinkage stress. This is consistent with the creep theory, that is, under the same shrinkage strain, the higher the elastic modulus, the greater the shrinkage stress, and the low modulus of solid waste concrete can alleviate this effect [173].

Soda residue can regulate the drying shrinkage of concrete through two paths: (1) Optimize the pore structure: Its hydration products can significantly improve the compactness of cement paste and effectively reduce the drying shrinkage, which is particularly obvious in the alkali-activated system. (2) Expansion compensation: The induced expansion products, such as AFt, provide volume compensation for drying shrinkage, but excessive mixing may increase the shrinkage risk due to the introduction of bubbles or the increase in evaporation water.

## 6. Research Gaps and Priority Directions

### 6.1. Long-Term Stability of Friedel’s Salt

Current research has identified key factors influencing the stability of Fs, such as pH [136], temperature, and the presence of sulfate ions [135]. However, systematic understanding of its long-term stability is still lacking: (1) Multi-factor coupling mechanisms remain unclear: Existing studies are predominantly single-factor laboratory tests, lacking investigations into the coupling effects of realistic conditions, such as carbonation–sulfate attack–wet–dry cycling–freeze–thaw cycles. When the carbonation front overlaps with the sulfate ingress zone, the acceleration or inhibition of the decomposition reaction of Fs currently lacks quantitative data. (2) Absence of phase-transition thermodynamic and kinetic databases: Fundamental thermodynamic data, such as the solubility equilibrium constants and Gibbs free energy of phase transitions of Fs at various temperatures (20 °C~80 °C) [174,175,176], pH values (9~13) [177], and Cl^−^/SO_4_^2−^ molar ratios [178], are extremely scarce, making it impossible to establish reliable thermodynamic prediction models. (3) Insufficient linkage between microstructural evolution and macroscopic property degradation: How the Cl^−^ released from Fs decomposition redistributes within the pore microstructure and how this process quantitatively correlates with the breakdown of the steel passive film remains a research gap [135,179].

### 6.2. Behavior of Free Chloride Versus Bound Chloride

The “immobilization–release” of chloride ions is central to evaluating the durability of SR concrete. However, quantitative research on its dynamic behavior is critically lacking: (1) Lack of systematic studies on chloride binding isotherms [180,181]: Chloride binding isotherm data for different SR contents, various composite cementitious systems (SR–GGBS [63], SR–FA [78], SR–SS [78]), and under various temperatures (5~60 °C) and ionic strengths are virtually non-existent. This makes it impossible to quantify the relative contributions of physical adsorption by C–S–H gel and chemical binding by AFm phases [182,183]. (2) Unclear critical thresholds for desorption behavior: The critical environmental conditions (critical pH, critical SO_4_^2−^ concentration) that trigger the desorption of bound chloride under scenarios such as carbonation-induced pH reduction [184], ion exchange due to sulfate ingress [135,185], or salt concentration during wet–dry cycles have not been systematically determined. (3) Absence of a coupled diffusion–binding–desorption kinetic model: Traditional Fick’s second law assumes that chloride binding is an instantaneous equilibrium linear process, which cannot describe the nonlinear binding and reversible decomposition behavior of Fs in SR systems [186]. There is an urgent need to develop mass-transfer kinetic models that couple chemical reactions.

### 6.3. Performance in Reinforced Concrete (RC)

The vast majority of current research remains at the level of paste or mortar, with very few studies addressing the long-term service behavior of real RC elements: (1) Unclear degradation mechanisms of RC elements in soda residue soil (SRS) environments: The chloride concentration in coastal SRS can be 2~3 times that of seawater [187]. When RC structure foundations or backfills consist of SRS, key issues such as the two/three-dimensional transport laws of chloride under unsaturated conditions and the spatiotemporal evolution of steel depassivation have not been systematically investigated. (2) Undetermined Critical Chloride Threshold Level (CCTL) for SR–containing concrete: Due to the high alkalinity and complex ionic composition introduced by SR, the pore solution chemistry differs significantly from that of conventional OPC concrete. Reliable experimental data on the CCTL for SR-containing systems are still lacking. (3) Absence of structural–level durability design methods: The establishment of service life prediction models for SR-containing RC elements, based on parameters such as chloride permeability coefficient, CCTL, and concrete cover thickness, represents a bridging gap connecting material research to engineering application.

### 6.4. Desalination Strategies and Pretreatment Processes

Desalination is the fundamental approach to eliminating the chloride risk of SR [188], but a technical solution suitable for large-scale implementation is yet to be developed: (1) Lack of low-energy, low-water-consumption dechlorination technologies: Traditional water washing is water-intensive and generates high-salinity wastewater, while electrokinetic methods are energy-intensive and slow. How to integrate technologies such as industrial waste heat utilization, CO_2_-assisted water washing, and selective electrodialysis to develop lower-cost, more environmentally friendly dechlorination processes remains a significant technical gap [189]. (2) Unclear mechanisms for preserving and regulating SR activity after desalination: Water washing or chemical desalination processes simultaneously leach out valuable active components such as Ca^2+^ and OH^−^, leading to a decrease in the cementitious activity or alkaline activation capacity of the treated SR. How to achieve “selective dechlorination” while retaining useful components is a technical challenge. (3) Lack of a holistic scheme for the resource utilization of desalination wastewater: NaCl and CaCl_2_ in the wash water are potential industrial salt resources. Current research focuses more on wastewater treatment than resource recovery, lacking an integrated “desalination–salt separation–water recycling–zero discharge” full-process technology.

By conducting principal analysis, performance comparison, cost accounting, and environmental assessment of mainstream technologies such as water washing and electrokinetic methods, combined with frontier explorations like multi-technology coupling and water resource recycling, it is expected that efficient pretreatment technologies possessing both economic viability and environmental friendliness can be developed, thereby fundamentally resolving the concerns posed by chloride ions.

### 6.5. Environmental Assessment of Pre-Treatment Processes

The evaluation dimensions of existing studies mostly focus on mechanical properties and short-term durability, lacking systematic assessments: (1) Gap in Life Cycle Assessment (LCA) models: For different pretreatment pathways (water washing [49], electrokinetic, CO_2_-assisted water washing [188]) and different end-use scenarios (alkali-activated cementitious materials [57], road base fillers [190], foamed concrete [144]), there is a lack of unified LCA models with clearly defined boundaries, making it impossible to quantify comprehensive environmental performance indicators such as Global Warming Potential (GWP) and Acidification Potential (AP); (2) Lack of data for Techno-Economic Analysis (TEA): A huge gap exists between cost estimates at the laboratory scale and actual operational costs at the pilot/industrial scale. There is a lack of complete cost accounting data based on real pilot/demonstration projects, especially regarding the hidden costs of wastewater treatment, equipment depreciation, labor costs, and transportation costs. (3) Absence of comparative assessments with conventional disposal pathways: There is a lack of systematic comparison, from a full life-cycle perspective, between the “pretreatment + building material application “pathway and the “direct stockpiling/landfilling + virgin raw material production” pathway, which is crucial for policy-making and industrial investment decisions.

### 6.6. Techno-Economic Feasibility at Scale

SR is typically used in combination with slag, fly ash, steel slag, etc., but the long-term durability behavior of multi-component systems is far more complex than that of single systems: (1) Unclear synergistic/antagonistic effects of multiple solid waste components: In multi-component systems such as SR–GGBS–FA, the quantitative relationships governing the interactions among the activity release rates of each component, the evolution of hydration products, and the pore structure development remain unclear [21,108]. (2) Lack of performance data under extreme service conditions: Long-term performance data under extreme conditions, such as freeze–thaw cycles, coupled carbonation–chloride attack, and microbiologically induced corrosion, are extremely scarce. (3) Absence of standardized accelerated testing methods: Current durability tests mostly employ single-factor accelerated methods, lacking standardized accelerated testing protocols that can effectively simulate multi-factor coupling effects, resulting in poor data comparability between different studies.

Furthermore, the long-term volumetric stability of SR-blended systems under coupled environmental exposures, including drying shrinkage, autogenous shrinkage, and the balance between expansive hydration products (AFt, Fs) and long-term dimensional stability, remains poorly characterized. Dedicated long-term deformation monitoring under varying humidity and temperature conditions is needed. Similarly, the durability of SR-blended materials under wet–dry cycling and freeze–thaw attack has rarely been investigated, and the absence of standardized multi-factor durability testing protocols represents a significant barrier to service life prediction.

## 7. Conclusions and Prospects

This study summarizes the application of soda residue in cementitious materials from the aspects of its physical and chemical properties, microstructure, and impact on cementitious materials. The main conclusions are as follows:

Physicochemical characteristics and pretreatment-dependent properties: Soda residue is an industrial by-product with a lightweight porous honeycomb structure (high porosity of ~60%, high specific surface area of 200–600 m^2^/kg), mainly composed of calcium salts (CaCO_3_, CaSO_4,_ etc.), with high alkalinity (pH > 10) and Cl^−^/Mg^2+^ enrichment characteristics. Its active components and pore structure can be directionally controlled through calcination, grinding, and chemical treatment. Each pretreatment method produces a distinct SR type that exhibits fundamentally different behavior in cementitious systems: untreated SR (RSR) functions primarily as an alkaline activator in alkali-activated systems; water-washed SR (WSR), with reduced chloride content, is more suitable for Portland cement systems; wet-ground SR (WMSR) provides nucleation seeding and micro-filling effects; and calcined SR (CSR) contributes enhanced pozzolanic reactivity.

In Portland cement systems at controlled dosages (typically <10% for untreated SR and <6% for wet-ground SR) under adequate curing conditions, SR refines the pore structure and densifies the matrix through physical filling, nucleation seeding, and chemical activation, promoting the transition from a loosely packed to a densified microstructure and strengthening particle interface bonding. In alkali-activated slag systems, SR serves additionally as an alkaline activator.

Coupled effect on workability and mechanical properties: When used at appropriate dosages (typically <10% in Portland cement systems, and SR:GGBS ≤ 16:84 in alkali-activated slag systems) and under adequate wet curing conditions, soda residue improves the compactness and early strength of concrete through the synergistic effect of physics and chemistry, In contrast, in systems employing untreated SR at higher replacement levels, its high water absorption and chloride salt crystallization characteristics lead to the deterioration of rheological properties, forming a coupling effect of mechanical enhancement and workability reduction, which is controlled by multiple factors such as dosage, gradation, pretreatment method and curing system.

Soda residue has a bidirectional effect on the durability of concrete. Positively, at controlled dosages in appropriately designed composite systems and under conditions that favor stable Fs formation, SR improves impermeability and crack resistance through chemical chlorine fixation, pore structure optimization, and internal curing functions. In the negative direction, excessive or improper mixing can cause chloride salt crystallization expansion, intensified sulfate corrosion, and steel corrosion risk. Durability optimization needs to be achieved through dosage control, mineral admixture compounding, and dechlorination treatment, combined with the use of high-efficiency water-reducing agents and wet curing to enhance durability. For any reinforced concrete application, the total chloride content must be verified to comply with the limits specified in relevant structural concrete design codes (e.g., ≤0.06–0.30% by mass of cement, depending on exposure class).

Adding an appropriate amount of soda residue to cementitious materials can improve their mechanical and durability properties, reduce its self shrinkage, but compared to industrial waste such as slag, fly ash, and steel slag, the large-scale application of soda residue faces bottlenecks such as performance fluctuations, high pretreatment costs, pollutant leaching risks, chloride salt and alkali–aggregate reactions, and potential expansion. Future research needs to focus on breakthroughs in three major directions: the development of soda residue composite high-strength cementitious materials, technological innovation in the preparation of solid waste-based cementitious materials, and collaborative modification technology for efficient dechlorination and desalination processes, ultimately achieving the industrialization goal of balancing performance, cost, and environmental coordination.

## Figures and Tables

**Table 1:** Basic physical properties of soda residue [16,21,22,23,24,25,26,27,28,29].

ρ (g/cm^3^)	SSA (m^2^/kg)	Moisture Content (%)	Plastic Limit (%)	Liquid Limit (%)	Particle Size (μm)
2.25~2.35 [16,22,25,26,27]	200~600 [16,21,23,25]	40~60 [16,21,23,25,29]	38~54 [24,28,29]	60~90 [24,28,29]	≤25 μm [21,25,26,29]

Note: ρ = particle density; SSA = specific surface area; all percentages reported in Table 1 (e.g., moisture content, plastic limit, liquid limit) represent mass percentages (% *w*/*w*).

**Table 3:** Comparative analysis of methods for soda residue modification.

Type	Regulatory Mechanism	Key Property Changes	Typical Application Scenarios
Raw	Bone	High Cl^−^, low reactivity; Complex composition; Low cost, simple process.	Clinker-free binder activator; S/S solidifying agent.
Washed	Dissolution– separation	Cl^−^ content significantly reduced; pH decreased; Effectively removes harmful ions.	Mineral admixture for cement concrete.
Wet-Milled	Mechanical comminution	Reduced particle size; Increased SSA; Enhanced reactivity; Uniform dispersion.	Cement early–strength agent; seeding, high-performance concrete.
Calcined	Thermal decomposition –phase transition	Formation of reactive CaO/Ca(OH)_2_; loosened structure; Greatly enhanced pozzolanic activity.	High–activity cementitious material component.
Chemically Modified	Chemical reaction, synergistic effect	Composition reconstruction; Multiple activation; Strongly designable performance; synergistic enhancement.	Alkali–calcium synergistically activated binders, specialty functional materials.

**Table 4:** The synergistic effect of the soda residue–single mineral admixture binary system.

Binary System	Synergistic Reaction with Soda Residue
SR–Fly ash	Pozzolanic effect: The inherent alkalinity of the soda residue effectively activates the reactive SiO_2_ within fly ash, facilitating the formation of cementitious hydration products [29]. Ion exchange reaction: In alkaline conditions, Ca^2+^ from soda residue participates in ion exchange with alkali metal ions (Na^+^, K^+^) on fly ash particle surfaces. This exchange facilitates depolymerization and dissolution of the fly ash vitreous matrix, releasing reactive Si^4+^ and Al^3+^ that form precursors for polymerization reactions [78]. Gel polymerization reaction: The released reactive ions undergo polycondensation to form oligomeric gels, which further polymerize into three–dimensional network gel phases (C–A–S–H, Sodium Aluminosilicate Hydrate(N–A–S–H). As key strength contributing phases, these gels densify the microstructure by pore filling, thereby enhancing overall compactness [79].
SR–GGBS	Alkali-activated reaction: Alkali activators (Na_2_O, K_2_O) derived from soda residue react with reactive SiO_2_ and Al_2_O_3_ in slag, generating hydraulic cementitious phases [30]. Cl^−^ solidification: Slag can combine with Cl^−^ from soda residues to form Fs, effectively immobilizing chloride. This process not only addresses the issues caused by high chloride concentration in soda residue but also improves the performance of the material [63]. Hydration promotion: Soda residue activates slag hydration, facilitating the formation of C–S–H, C–A–S–H, and Fs. Supplementary Ca(OH)_2_ and CaCl_2_ from soda residue elevate Ca^2+^ concentration in the system, accelerating hydration kinetics and promoting C–S–H gel nucleation and growth [62].
SR–SS	Complexation reaction: During later hydration stages, Calcium (Alumino–) Silicate Hydrate(C–(A)–S–H) gels integrate with soda residue, undergoing microstructural densification that refines pore networks. This cohesive transformation enhances compactness and structural integrity, ultimately elevating mechanical performance and durability [80]. AFt Formation: Calcium species from soda residue enable early–stage AFt formation. Deposited on steel slag particles, AFt concurrently modulates hydration kinetics through surface inhibition while functioning as a structural framework to densify the matrix. This dual mechanism refines pore architecture and significantly reduces drying shrinkage [81]. Alkali-activated reaction: The inherent alkalinity of soda residue provides OH^−^ ions that effectively activate latent reactive minerals in steel slag (C_3_S, C_2_S). This activation accelerates mineral dissolution and subsequent hydration, generating strength-enhancing C–S–H gel to optimize cementitious performance [82].

**Table 5:** The synergistic effect of soda residue–multi-mineral admixture system.

Multi-Component System	Synergistic Reaction with Soda Residue
SR-Slag-Gypsum	The hydration products, including C–S–H, AFt, and Fs, form a dense three-dimensional network structure. Concurrently, gel phases cohesively integrate unreacted particles within this network while infilling interstitial spaces, inducing microstructural densification that elevates concrete compressive strength [56].
SR-GGBS-iron tailings	Primary cementitious phases C–S–H, C–A–S–H, and Fs exhibit progressive accumulation with extended curing, ensuring strength development from early to mature stages in the binder system [57].
SR-Carbide slag-red mud–Fly ash	The alkaline environment derived from carbide slag, soda residue, and red mud drives congruent dissolution of reactive phases in fly ash and red mud. This process facilitates the formation of a cohesive three-dimensional network comprising N–A–S–H, C–(A)–S–H, and Fs, significantly enhancing early strength development. Concurrently, Cl^−^ from soda residue is chemically immobilized by red mud components, mitigating permeability risks through chloride penetration [83].

**Table 7:** Chloride content limits in concrete are specified by different design codes.

Code/Standard	Exposure Class	Chloride Limit (%)
China Code for Design of Concrete Structures (GB 50010 [127])	Class I (Indoor dry environment)	≤0.30%
	Class IIa (Humid outdoor environment)	≤0.20%
	Class IIIa (Marine salt spray zone)	≤0.10%
USA Building Code Requirements for Structural Concrete (ACI 318 [128])	–	≤0.07% (Prestressed) /0.15% (Non-prestressed)
Europe Design of Concrete Structures (EN 1992-1-1 [129])	XD1/XC4 (Moderate corrosion)	≤0.40%

Note: Chloride limits are expressed as percent by mass of cementitious binder (% *w*/*w*). In the ACI 318 row, “≤0.07% (Prestressed)/0.15% (Non-prestressed)” means “≤0.07% for prestressed concrete and ≤0.15% for non-prestressed concrete.”

**Table 8:** CCTL in different studies.

Concrete Type	CCTL (% by Weight of Cement)	Test Method
Conventional concrete (normal) [130]	0.44%	RCI accelerated test
High-volume fly ash concrete [130]	0.069%	RCI accelerated test
Marine concrete (marine environment) [131]	0.07–0.70%	Review, influenced by multiple factors
Conventional concrete (literature values) [131]	0.20–1.0%	Review, wide fluctuation range
Concrete affected by sulfates [133]	Reduced	Chloride threshold lowered due to sulfate presence

## Data Availability

No new data were created or analyzed in this study. Data sharing is not applicable to this article.
